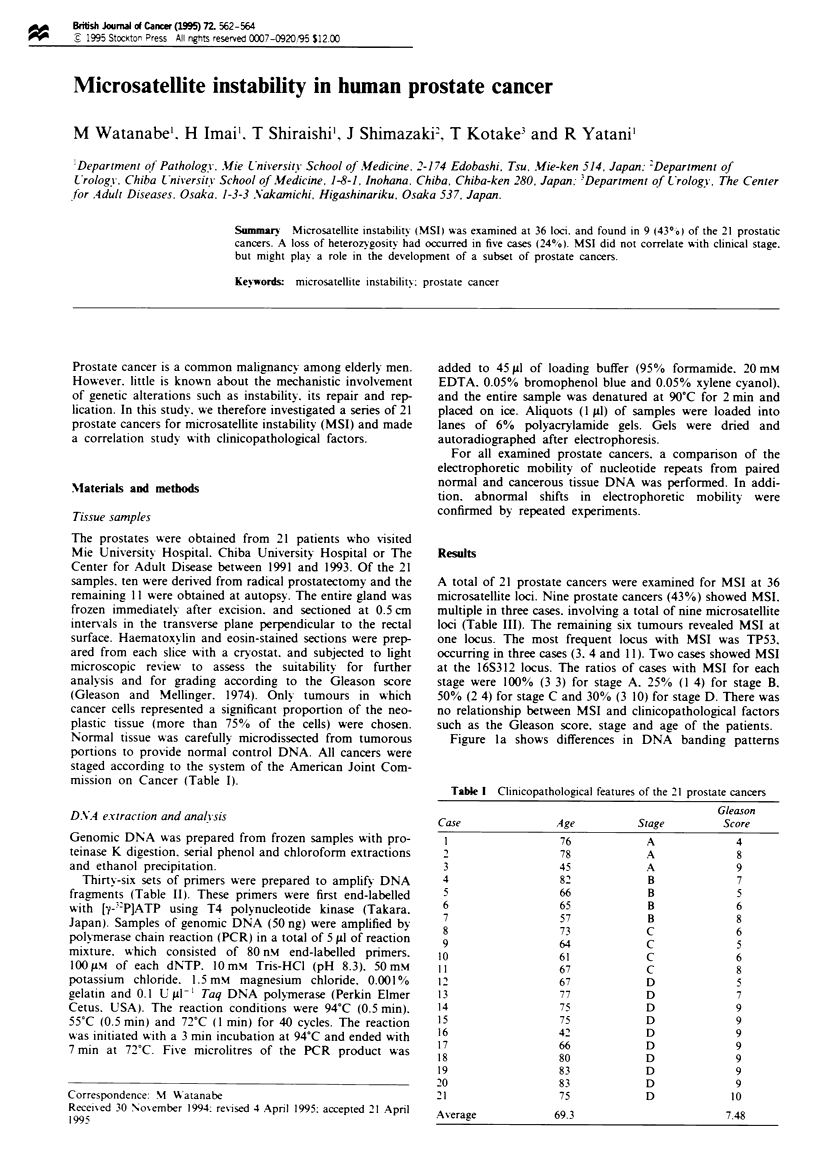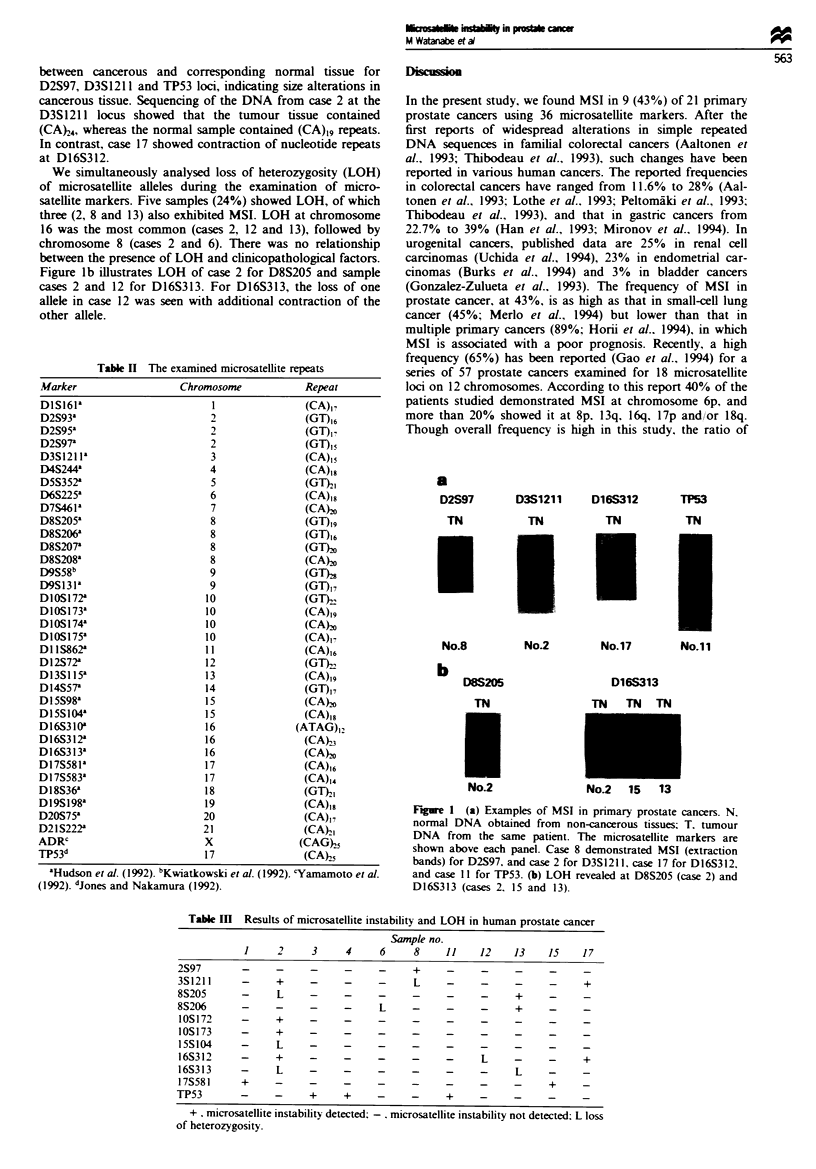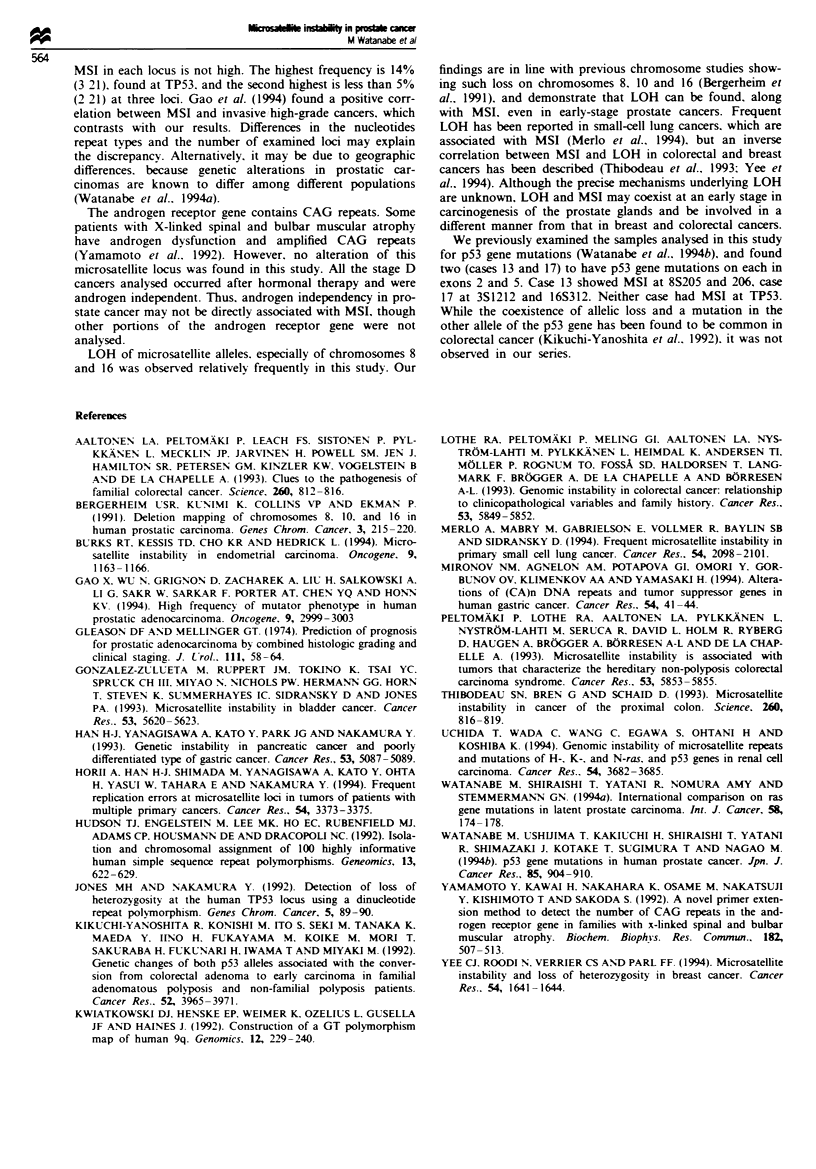# Microsatellite instability in human prostate cancer.

**DOI:** 10.1038/bjc.1995.374

**Published:** 1995-09

**Authors:** M. Watanabe, H. Imai, T. Shiraishi, J. Shimazaki, T. Kotake, R. Yatani

**Affiliations:** Department of Pathology, Mie University School of Medicine, Japan.

## Abstract

**Images:**


British Journal of Cancer (1995) 72, 562-564

© 1995 Stockton Press All rights reserved 0007-0920/95 $12.00

### Microsatellite instability in human prostate cancer

M Watanabe[1], H Imai[1], T Shiraishi[1], J Shimazaki[2], T Kotake[3] and R Yatani[1]

[1]Department of Pathology, Mie University School of Medicine, 2-174 Edobashi, Tsu, Mie-ken 514, Japan; [2]Department of Urology, Chiba University School of Medicine, 1-8-1, Inohana, Chiba, Chiba-ken 280, Japan; [3]Department of Urology, The Center for Adult Diseases, Osaka, 1-3-3 Nakamichi, Higashinariku, Osaka 537, Japan.

**Summary** Microsatellite instability (MSI) was examined at 36 loci, and found in 9 (43%) of the 21 prostatic cancers. A loss of heterozygosity had occurred in five cases (24%). MSI did not correlate with clinical stage, but might play a role in the development of a subset of prostate cancers.

**Keywords:** microsatellite instability; prostate cancer

Prostate cancer is a common malignancy among elderly men. However, little is known about the mechanistic involvement of genetic alterations such as instability, its repair and replication. In this study, we therefore investigated a series of 21 prostate cancers for microsatellite instability (MSI) and made a correlation study with clinicopathological factors.

### Materials and methods

### Tissue samples

The prostates were obtained from 21 patients who visited Mie University Hospital, Chiba University Hospital or The Center for Adult Disease between 1991 and 1993. Of the 21 samples, ten were derived from radical prostatectomy and the remaining 11 were obtained at autopsy. The entire gland was frozen immediately after excision, and sectioned at 0.5 cm intervals in the transverse plane perpendicular to the rectal surface. Haematoxylin and eosin-stained sections were prepared from each slice with a cryostat, and subjected to light microscopic review to assess the suitability for further analysis and for grading according to the Gleason score (Gleason and Mellinger, 1974). Only tumours in which cancer cells represented a significant proportion of the neoplastic tissue (more than 75% of the cells) were chosen. Normal tissue was carefully microdissected from tumorous portions to provide normal control DNA. All cancers were staged according to the system of the American Joint Commission on Cancer (Table I).

### DNA extraction and analysis

Genomic DNA was prepared from frozen samples with proteinase K digestion, serial phenol and chloroform extractions and ethanol precipitation.

Thirty-six sets of primers were prepared to amplify DNA fragments (Table II). These primers were first end-labelled with [$\gamma$-$^{32}$P]ATP using T4 polynucleotide kinase (Takara, Japan). Samples of genomic DNA (50 ng) were amplified by polymerase chain reaction (PCR) in a total of 5 µl of reaction mixture, which consisted of 80 nM end-labelled primers, 100 µM of each dNTP, 10 mM Tris-HCl (pH 8.3), 50 mM potassium chloride, 1.5 mM magnesium chloride, 0.001% gelatin and 0.1 U $\mu l^{-1}$ *Taq* DNA polymerase (Perkin Elmer Cetus, USA). The reaction conditions were 94°C (0.5 min), 55°C (0.5 min) and 72°C (1 min) for 40 cycles. The reaction was initiated with a 3 min incubation at 94°C and ended with 7 min at 72°C. Five microlitres of the PCR product was added to 45 µl of loading buffer (95% formamide, 20 mM EDTA, 0.05% bromophenol blue and 0.05% xylene cyanol), and the entire sample was denatured at 90°C for 2 min and placed on ice. Aliquots (1 µl) of samples were loaded into lanes of 6% polyacrylamide gels. Gels were dried and autoradiographed after electrophoresis.

For all examined prostate cancers, a comparison of the electrophoretic mobility of nucleotide repeats from paired normal and cancerous tissue DNA was performed. In addition, abnormal shifts in electrophoretic mobility were confirmed by repeated experiments.

### Results

A total of 21 prostate cancers were examined for MSI at 36 microsatellite loci. Nine prostate cancers (43%) showed MSI, multiple in three cases, involving a total of nine microsatellite loci (Table III). The remaining six tumours revealed MSI at one locus. The most frequent locus with MSI was TP53, occurring in three cases (3, 4 and 11). Two cases showed MSI at the 16S312 locus. The ratios of cases with MSI for each stage were 100% (3/3) for stage A, 25% (1/4) for stage B, 50% (2/4) for stage C and 30% (3/10) for stage D. There was no relationship between MSI and clinicopathological factors such as the Gleason score, stage and age of the patients.

Figure 1a shows differences in DNA banding patterns

**Table I** Clinicopathological features of the 21 prostate cancers

| *Case* | *Age* | *Stage* | *Gleason Score* |
|---|---|---|---|
| 1 | 76 | A | 4 |
| 2 | 78 | A | 8 |
| 3 | 45 | A | 9 |
| 4 | 82 | B | 7 |
| 5 | 66 | B | 5 |
| 6 | 65 | B | 6 |
| 7 | 57 | B | 8 |
| 8 | 73 | C | 6 |
| 9 | 64 | C | 5 |
| 10 | 61 | C | 6 |
| 11 | 67 | C | 8 |
| 12 | 67 | D | 5 |
| 13 | 77 | D | 7 |
| 14 | 75 | D | 9 |
| 15 | 75 | D | 9 |
| 16 | 42 | D | 9 |
| 17 | 66 | D | 9 |
| 18 | 80 | D | 9 |
| 19 | 83 | D | 9 |
| 20 | 83 | D | 9 |
| 21 | 75 | D | 10 |
| Average | 69.3 | | 7.48 |

Correspondence: M Watanabe

Received 30 November 1994; revised 4 April 1995; accepted 21 April 1995

between cancerous and corresponding normal tissue for D2S97, D3S1211 and TP53 loci, indicating size alterations in cancerous tissue. Sequencing of the DNA from case 2 at the D3S1211 locus showed that the tumour tissue contained $(CA)_{24}$, whereas the normal sample contained $(CA)_{19}$ repeats. In contrast, case 17 showed contraction of nucleotide repeats at D16S312.

We simultaneously analysed loss of heterozygosity (LOH) of microsatellite alleles during the examination of microsatellite markers. Five samples (24%) showed LOH, of which three (2, 8 and 13) also exhibited MSI. LOH at chromosome 16 was the most common (cases 2, 12 and 13), followed by chromosome 8 (cases 2 and 6). There was no relationship between the presence of LOH and clinicopathological factors. Figure 1b illustrates LOH of case 2 for D8S205 and sample cases 2 and 12 for D16S313. For D16S313, the loss of one allele in case 12 was seen with additional contraction of the other allele.

**Table II** The examined microsatellite repeats

| *Marker* | *Chromosome* | *Repeat* |
|---|---|---|
| D1S161[a] | 1 | $(CA)_{17}$ |
| D2S93[a] | 2 | $(GT)_{16}$ |
| D2S95[a] | 2 | $(GT)_{17}$ |
| D2S97[a] | 2 | $(GT)_{15}$ |
| D3S1211[a] | 3 | $(CA)_{15}$ |
| D4S244[a] | 4 | $(CA)_{18}$ |
| D5S352[a] | 5 | $(GT)_{21}$ |
| D6S225[a] | 6 | $(CA)_{18}$ |
| D7S461[a] | 7 | $(CA)_{20}$ |
| D8S205[a] | 8 | $(GT)_{19}$ |
| D8S206[a] | 8 | $(GT)_{16}$ |
| D8S207[a] | 8 | $(GT)_{20}$ |
| D8S208[a] | 8 | $(CA)_{20}$ |
| D9S58[b] | 9 | $(GT)_{28}$ |
| D9S131[a] | 9 | $(GT)_{17}$ |
| D10S172[a] | 10 | $(GT)_{22}$ |
| D10S173[a] | 10 | $(CA)_{19}$ |
| D10S174[a] | 10 | $(CA)_{20}$ |
| D10S175[a] | 10 | $(CA)_{17}$ |
| D11S862[a] | 11 | $(CA)_{16}$ |
| D12S72[a] | 12 | $(GT)_{22}$ |
| D13S115[a] | 13 | $(CA)_{19}$ |
| D14S57[a] | 14 | $(GT)_{17}$ |
| D15S98[a] | 15 | $(CA)_{20}$ |
| D15S104[a] | 15 | $(CA)_{18}$ |
| D16S310[a] | 16 | $(ATAG)_{12}$ |
| D16S312[a] | 16 | $(CA)_{23}$ |
| D16S313[a] | 16 | $(CA)_{20}$ |
| D17S581[a] | 17 | $(CA)_{16}$ |
| D17S583[a] | 17 | $(CA)_{14}$ |
| D18S36[a] | 18 | $(GT)_{21}$ |
| D19S198[a] | 19 | $(CA)_{18}$ |
| D20S75[a] | 20 | $(CA)_{17}$ |
| D21S222[a] | 21 | $(CA)_{21}$ |
| ADR[c] | X | $(CAG)_{25}$ |
| TP53[d] | 17 | $(CA)_{25}$ |

[a]Hudson *et al.* (1992). [b]Kwiatkowski *et al.* (1992). [c]Yamamoto *et al.* (1992). [d]Jones and Nakamura (1992).

### Discussion

In the present study, we found MSI in 9 (43%) of 21 primary prostate cancers using 36 microsatellite markers. After the first reports of widespread alterations in simple repeated DNA sequences in familial colorectal cancers (Aaltonen *et al.*, 1993; Thibodeau *et al.*, 1993), such changes have been reported in various human cancers. The reported frequencies in colorectal cancers have ranged from 11.6% to 28% (Aaltonen *et al.*, 1993; Lothe *et al.*, 1993; Peltomäki *et al.*, 1993; Thibodeau *et al.*, 1993), and that in gastric cancers from 22.7% to 39% (Han *et al.*, 1993; Mironov *et al.*, 1994). In urogenital cancers, published data are 25% in renal cell carcinomas (Uchida *et al.*, 1994), 23% in endometrial carcinomas (Burks *et al.*, 1994) and 3% in bladder cancers (Gonzalez-Zulueta *et al.*, 1993). The frequency of MSI in prostate cancer, at 43%, is as high as that in small-cell lung cancer (45%; Merlo *et al.*, 1994) but lower than that in multiple primary cancers (89%; Horii *et al.*, 1994), in which MSI is associated with a poor prognosis. Recently, a high frequency (65%) has been reported (Gao *et al.*, 1994) for a series of 57 prostate cancers examined for 18 microsatellite loci on 12 chromosomes. According to this report 40% of the patients studied demonstrated MSI at chromosome 6p, and more than 20% showed it at 8p, 13q, 16q, 17p and/or 18q. Though overall frequency is high in this study, the ratio of

**Figure 1** (**a**) Examples of MSI in primary prostate cancers. N, normal DNA obtained from non-cancerous tissues; T, tumour DNA from the same patient. The microsatellite markers are shown above each panel. Case 8 demonstrated MSI (extraction bands) for D2S97, and case 2 for D3S1211, case 17 for D16S312, and case 11 for TP53. (**b**) LOH revealed at D8S205 (case 2) and D16S313 (cases 2, 15 and 13).

**Table III** Results of microsatellite instability and LOH in human prostate cancer

| | *Sample no.* | | | | | | | | | | |
|---|---|---|---|---|---|---|---|---|---|---|---|
| | *1* | *2* | *3* | *4* | *6* | *8* | *11* | *12* | *13* | *15* | *17* |
| 2S97 | – | – | – | – | – | + | – | – | – | – | – |
| 3S1211 | – | + | – | – | – | L | – | – | – | – | + |
| 8S205 | – | L | – | – | – | – | – | – | + | – | – |
| 8S206 | – | – | – | – | L | – | – | – | + | – | – |
| 10S172 | – | + | – | – | – | – | – | – | – | – | – |
| 10S173 | – | + | – | – | – | – | – | – | – | – | – |
| 15S104 | – | L | – | – | – | – | – | – | – | – | – |
| 16S312 | – | + | – | – | – | – | – | L | – | – | + |
| 16S313 | – | L | – | – | – | – | – | – | L | – | – |
| 17S581 | + | – | – | – | – | – | – | – | – | + | – |
| TP53 | – | – | + | + | – | – | + | – | – | – | – |

+, microsatellite instability detected; –, microsatellite instability not detected; L loss of heterozygosity.

MSI in each locus is not high. The highest frequency is 14% (3 21). found at TP53. and the second highest is less than 5% (2 21) at three loci. Gao *et al.* (1994) found a positive correlation between MSI and invasive/high-grade cancers, which contrasts with our results. Differences in the nucleotides repeat types and the number of examined loci may explain the discrepancy. Alternatively, it may be due to geographic differences, because genetic alterations in prostatic carcinomas are known to differ among different populations (Watanabe *et al.*, 1994*a*).

The androgen receptor gene contains CAG repeats. Some patients with X-linked spinal and bulbar muscular atrophy have androgen dysfunction and amplified CAG repeats (Yamamoto *et al.*, 1992). However, no alteration of this microsatellite locus was found in this study. All the stage D cancers analysed occurred after hormonal therapy and were androgen independent. Thus, androgen independency in prostate cancer may not be directly associated with MSI, though other portions of the androgen receptor gene were not analysed.

LOH of microsatellite alleles, especially of chromosomes 8 and 16 was observed relatively frequently in this study. Our findings are in line with previous chromosome studies showing such loss on chromosomes 8, 10 and 16 (Bergerheim *et al.*, 1991), and demonstrate that LOH can be found, along with MSI, even in early-stage prostate cancers. Frequent LOH has been reported in small-cell lung cancers, which are associated with MSI (Merlo *et al.*, 1994), but an inverse correlation between MSI and LOH in colorectal and breast cancers has been described (Thibodeau *et al.*, 1993; Yee *et al.*, 1994). Although the precise mechanisms underlying LOH are unknown, LOH and MSI may coexist at an early stage in carcinogenesis of the prostate glands and be involved in a different manner from that in breast and colorectal cancers.

We previously examined the samples analysed in this study for p53 gene mutations (Watanabe *et al.*, 1994*b*), and found two (cases 13 and 17) to have p53 gene mutations on each in exons 2 and 5. Case 13 showed MSI at 8S205 and 206, case 17 at 3S1212 and 16S312. Neither case had MSI at TP53. While the coexistence of allelic loss and a mutation in the other allele of the p53 gene has been found to be common in colorectal cancer (Kikuchi-Yanoshita *et al.*, 1992), it was not observed in our series.

### References

AALTONEN LA, PELTOMÄKI P, LEACH FS, SISTONEN P, PYLKKÄNEN L, MECKLIN JP, JARVINEN H, POWELL SM, JEN J, HAMILTON SR, PETERSEN GM, KINZLER KW, VOGELSTEIN B AND DE LA CHAPELLE A. (1993). Clues to the pathogenesis of familial colorectal cancer. *Science*, **260**, 812–816.

BERGERHEIM USR, KUNIMI K, COLLINS VP AND EKMAN P. (1991). Deletion mapping of chromosomes 8, 10, and 16 in human prostatic carcinoma. *Genes Chrom. Cancer*, **3**, 215–220.

BURKS RT, KESSIS TD, CHO KR AND HEDRICK L. (1994). Microsatellite instability in endometrial carcinoma. *Oncogene*, **9**, 1163–1166.

GAO X, WU N, GRIGNON D, ZACHAREK A, LIU H, SALKOWSKI A, LI G, SAKR W, SARKAR F, PORTER AT, CHEN YQ AND HONN KV. (1994). High frequency of mutator phenotype in human prostatic adenocarcinoma. *Oncogene*, **9**, 2999–3003

GLEASON DF AND MELLINGER GT. (1974). Prediction of prognosis for prostatic adenocarcinoma by combined histologic grading and clinical staging. *J. Urol.*, **111**, 58–64.

GONZALEZ-ZULUETA M, RUPPERT JM, TOKINO K, TSAI YC, SPRUCK CH III, MIYAO N, NICHOLS PW, HERMANN GG, HORN T, STEVEN K, SUMMERHAYES IC, SIDRANSKY D AND JONES PA. (1993). Microsatellite instability in bladder cancer. *Cancer Res.*, **53**, 5620–5623.

HAN H-J, YANAGISAWA A, KATO Y, PARK JG AND NAKAMURA Y. (1993). Genetic instability in pancreatic cancer and poorly differentiated type of gastric cancer. *Cancer Res.*, **53**, 5087–5089.

HORII A, HAN H-J, SHIMADA M, YANAGISAWA A, KATO Y, OHTA H, YASUI W, TAHARA E AND NAKAMURA Y. (1994). Frequent replication errors at microsatellite loci in tumors of patients with multiple primary cancers. *Cancer Res.*, **54**, 3373–3375.

HUDSON TJ, ENGELSTEIN M, LEE MK, HO EC, RUBENFIELD MJ, ADAMS CP, HOUSMANN DE AND DRACOPOLI NC. (1992). Isolation and chromosomal assignment of 100 highly informative human simple sequence repeat polymorphisms. *Geneomics*, **13**, 622–629.

JONES MH AND NAKAMURA Y. (1992). Detection of loss of heterozygosity at the human TP53 locus using a dinucleotide repeat polymorphism. *Genes Chrom. Cancer*, **5**, 89–90.

KIKUCHI-YANOSHITA R, KONISHI M, ITO S, SEKI M, TANAKA K, MAEDA Y, IINO H, FUKAYAMA M, KOIKE M, MORI T, SAKURABA H, FUKUNARI H, IWAMA T AND MIYAKI M. (1992). Genetic changes of both p53 alleles associated with the conversion from colorectal adenoma to early carcinoma in familial adenomatous polyposis and non-familial polyposis patients. *Cancer Res.*, **52**, 3965–3971.

KWIATKOWSKI DJ, HENSKE EP, WEIMER K, OZELIUS L, GUSELLA JF AND HAINES J. (1992). Construction of a GT polymorphism map of human 9q. *Genomics*, **12**, 229–240.

LOTHE RA, PELTOMÄKI P, MELING GI, AALTONEN LA, NYSTRÖM-LAHTI M, PYLKKÄNEN L, HEIMDAL K, ANDERSEN TI, MÖLLER P, ROGNUM TO, FOSSÅ SD, HALDORSEN T, LANGMARK F, BRÖGGER A, DE LA CHAPELLE A AND BÖRRESEN A-L. (1993). Genomic instability in colorectal cancer: relationship to clinicopathological variables and family history. *Cancer Res.*, **53**, 5849–5852.

MERLO A, MABRY M, GABRIELSON E, VOLLMER R, BAYLIN SB AND SIDRANSKY D. (1994). Frequent microsatellite instability in primary small cell lung cancer. *Cancer Res.*, **54**, 2098–2101.

MIRONOV NM, AGNELON AM, POTAPOVA GI, OMORI Y, GORBUNOV OV, KLIMENKOV AA AND YAMASAKI H. (1994). Alterations of (CA)n DNA repeats and tumor suppressor genes in human gastric cancer. *Cancer Res.*, **54**, 41–44.

PELTOMÄKI P, LOTHE RA, AALTONEN LA, PYLKKÄNEN L, NYSTRÖM-LAHTI M, SERUCA R, DAVID L, HOLM R, RYBERG D, HAUGEN A, BRÖGGER A, BÖRRESEN A-L AND DE LA CHAPELLE A. (1993). Microsatellite instability is associated with tumors that characterize the hereditary non-polyposis colorectal carcinoma syndrome. *Cancer Res.*, **53**, 5853–5855.

THIBODEAU SN, BREN G AND SCHAID D. (1993). Microsatellite instability in cancer of the proximal colon. *Science*, **260**, 816–819.

UCHIDA T, WADA C, WANG C, EGAWA S, OHTANI H AND KOSHIBA K. (1994). Genomic instability of microsatellite repeats and mutations of H-, K-, and N-*ras*, and p53 genes in renal cell carcinoma. *Cancer Res.*, **54**, 3682–3685.

WATANABE M, SHIRAISHI T, YATANI R, NOMURA AMY AND STEMMERMANN GN. (1994*a*). International comparison on ras gene mutations in latent prostate carcinoma. *Int. J. Cancer*, **58**, 174–178.

WATANABE M, USHIJIMA T, KAKIUCHI H, SHIRAISHI T, YATANI R, SHIMAZAKI J, KOTAKE T, SUGIMURA T AND NAGAO M. (1994*b*). p53 gene mutations in human prostate cancer. *Jpn. J. Cancer Res.*, **85**, 904–910.

YAMAMOTO Y, KAWAI H, NAKAHARA K, OSAME M, NAKATSUJI Y, KISHIMOTO T AND SAKODA S. (1992). A novel primer extension method to detect the number of CAG repeats in the androgen receptor gene in families with x-linked spinal and bulbar muscular atrophy. *Biochem. Biophys. Res. Commun.*, **182**, 507–513.

YEE CJ, ROODI N, VERRIER CS AND PARL FF. (1994). Microsatellite instability and loss of heterozygosity in breast cancer. *Cancer Res.*, **54**, 1641–1644.